# *Planococcus circulans* sp. nov., A Novel Bacterium Isolated from Kubuqi Desert Soil

**DOI:** 10.3390/microorganisms14010231

**Published:** 2026-01-19

**Authors:** Siqi Cui, Siyue Zhang, Ya Chen, Yuhua Xin, Jie Du, Weiwei Ping, Pengze Bai, Jianli Zhang

**Affiliations:** 1 Key Laboratory of Molecular Medicine and Biotherapy, School of Life Science, Beijing Institute of Technology, Beijing 100081, China; candy9797979797@163.com (S.C.); zhangsy0147@163.com (S.Z.); chenya111@foxmail.com (Y.C.); dj535339752@outlook.com (J.D.); 18810706117@163.com (W.P.); 13051705965@163.com (P.B.); 2 China General Microbiological Culture Collection Center, Institute of Microbiology, Chinese Academy of Sciences, Beijing 100101, China; xinyh@im.ac.cn

**Keywords:** *Planococcus circulans*, polyphasic taxonomy, desert soil, salt tolerant, new taxa

## Abstract

A novel bacterial strain, designated as 4-30^T^, was isolated from a soil sample collected from the Kubuqi Desert in Inner Mongolia, northern China. The isolate was a Gram-stain-positive, aerobic, motile, and coccus-shaped bacterium, and its colonies were circular, opaque, convex, smooth, and orange-pigmented on Luria–Bertani agar. Phylogenetic analysis based on 16S rRNA gene sequences showed that strain 4-30^T^ belonged to the genus *Planococcus*. Growth occurred at 4–38 °C (optimum, 25–28 °C), pH 6.0–11.0 (optimum, pH 9.0), and in 0–10% (*w*/*v*) NaCl (optimum, 1%). Strain 4-30^T^ contained iso-C_14:0_, anteiso-C_15:0_, C_16:1_ *ω*7*c* alcohol, and iso-C_16:0_ as major cellular fatty acids (>10%) and MK-7 and MK-8 as predominant menaquinones. Its polar lipid profile consisted of diphosphatidylglycerol, phosphatidylglycerol, phosphatidylethanolamine, and two unidentified polar lipids. The genomic DNA G+C content was 45.9%. The average nucleotide identity (ANI) values between strain 4-30^T^ and the closely related species were relatively low (ANIm < 85.6%, ANIb < 82.9% and OrthoANIu < 83.3%), and the digital DNA–DNA hybridization (dDDH) between strain 4-30^T^ and type strains of the genus *Planococcus* were 20.0–26.7%. Based on phylogenetic, genotypic, chemotaxonomic, and phenotypic analyses, strain 4-30^T^ is considered to represent a novel species of the genus *Planococcus*, for which the name *Planococcus circulans* sp. nov. is proposed. The type strain is 4-30^T^ (=CDMCC 1.2409^T^ = KCTC 43405^T^).

## 1. Introduction

The genus *Planococcus* within the family *Caryophanaceae* was first proposed by Migula (1894) to accommodate aerobic, Gram-stain-positive, and motile bacteria and the description of its taxon was amended many times according to the differences in physiological and biochemical characteristics, menaquinone profile, fatty acid composition, and DNA G+C content, primarily driven by advances in systematic methodologies [1]. The changes to the description of the genus *Planocococcus* were mainly based on physiological, biochemical, and molecular characteristics. In the early stages, it relied mainly on physiological and biochemical characteristics. Later, with the development of technology, chemical taxonomy methods were adopted, such as measuring predominant menaquinones, polar lipid profile, and cellular fatty acid composition to improve genus boundaries and distinguish them from the closely related genera. Then, developments in molecular biology and the widespread use of 16S rRNA gene sequence analysis led to the transfer of several species from *Planocococcus* to other genera. For example, five bacterial species originally allocated to the genus *Planococcus*, namely *Planococcus okeanokoites* (2001) [1,2], *Planococcus mcmeekinii* (2001) [2,3], *Planococcus alkanoclasticus* (2005) [4,5], *Planococcus psychrophilus* (2005) [5,6], and *Planococcus stackebrandtii* (2009) [7,8], were transferred to a newly proposed genus *Planomicrobium*, containing the signature nucleotides C and G in the 16S rRNA gene sequences at positions 183 and 190, and *Planococcus halophilus* had been reclassified to *Marinococcus halophilus*. At present, whole-genome sequences provide clear criteria for species classification within this genus, which can provide an accurate description of the *Planocococcus* genus. In addition, the *Planocococcus* genus can usually produce a variety of natural active compounds, leading it to receive extensive research interest for its broad stability and functionality under hard environmental conditions [9]. The genus *Planococcus* comprises 34 species with validly published and correct names (http://www.bacterio.net/planococcus.html, accessed on 6 December 2025); the type species is *Planococcus citreus* [10]. Members of the genus *Planococcus* have strong resistance to environmental stress, such as high salt concentration and low temperature. Some species within this genus can even grow at temperatures below −10 °C. A remarkable example is the extreme sub-zero-adapted *Planococcus halocryophilus*, which can actively grow and reproduce at −15 °C and exhibits exceptional tolerance to both cold and high salinity [11]. The genus *Planococcus* can widely exist in various ecological environments, such as desert [12], soil [13], sea [14], spring [15], and even in Antarctica [16].

The Kubuqi Desert is located in the north of the ridge of the Ordos Plateau in Inner Mongolia, China. It is characterized by an arid climate (annual precipitation is about 250~300 mm, but annual evaporation is 2100~2700 mm), extreme temperatures (ranging from −10 °C in winter to 40 °C in summer), high soil salinity, strong ultraviolet radiation, and nutrient-poor soil (soil organic matter content can be less than 0.5 %). As a result, the animal and plant resources in the habitat are scarce, and in this context, microorganisms become the main driving force of material metabolism and energy cycle in the ecosystem, playing a significant role in the fixation of organic substances and the composition of soil structure. Their persistence is sustained by inputs of water (from rare rainfall or dew) and minimal organic carbon supplies from decaying plant litter and root exudation, or atmospheric deposition. In addition, the soil microbial biomass is the active part of soil organic matter and a source of available nutrients, and photoautotrophic and chemoautotrophic bacteria are responsible for the majority of biological carbon fixation. Simultaneously, biological nitrogen fixation caused by nitrogen-fixing bacteria associated with soil crusts or plant rhizospheres represents a critical nitrogen input into these nitrogen-poor soils. Beyond biogeochemistry, these microorganisms play a foundational role in soil stabilization through the formation of biological soil crusts. Therefore, microbiomes in the Kubuqi Desert are not merely survivors but essential engineers of ecosystem function and structure. During an investigation of the microbial diversity of the Kubuqi Desert, we obtained and described a novel member of the genus *Planococcus*, designated strain 4-30^T^. The taxonomic characterization was determined, and this was crucial for our understanding of desert soil-related bacterial communities, as well as understanding potential biotechnological applications and their mechanism of extreme environmental adaptation. Based on the results from the polyphasic taxonomic study presented, strain 4-30^T^ was characterized and classified as a novel species of the genus *Planococcus*. The bacteria successfully isolated from the Kubuqi Desert, like *Planococcus circulans* strain 4-30^T^ in this study, are expected to possess characteristics about tolerance to salt, radiation, and drought, reflecting direct adaptation to these prevalent stresses. However, previous studies in this area have primarily focused on the isolation and phenotypic characterization of halotolerant strains, leaving their molecular adaptive mechanisms to high salinity largely unexplored. To address this gap, we not only isolated a novel halotolerant strain 4-30^T^ from the Kubuqi Desert, but also conducted whole-genome sequencing analysis to identify key genes and pathways involved in its adaptation. The accurate taxonomic description of this halophilic *Planococcus* strain establishes a clear reference for future studies. This work provides a foundation for subsequent genomic and mechanistic investigations aimed at understanding its salt tolerance at the genetic level. Identifying and characterizing such strains is a crucial first step toward exploring their potential ecological roles and adaptation mechanisms in hypersaline environments and evaluating their possible industrial applications. In addition, most studies on desert microbiomes have focused on dominant phyla such as Actinobacteria and Cyanobacteria, and the genus *Planococcus* (belonging to the Firmicutes) remains remarkably underrepresented in culture collections from extreme arid deserts. Thus, the specific physiological characteristics and genomic foundations that enable *Planococcus* species to colonize and adapt to the combined stresses of salinity, drought, and oligotrophy in desert ecosystems are largely unknown. This limits our understanding of the full functional diversity and adaptive strategies within desert soil communities.

The study can help to form a better understanding of the ecological roles of *Planococcus* species in hypersaline habitats. In addition, strain 4-30^T^ contained macrolide antibiotic resistance genes, tetracycline antibiotic resistance genes, and fluoroquinolone antibiotic resistance genes, and more studies about whether it has antibiotic biodegradation capability to control antibiotic pollution could be conducted to develop a method for the biodegradation of antibiotics.

## 2. Materials and Methods

### 2.1. Bacterial Isolation and Cultivation

Strain 4-30^T^ was isolated from a soil sample collected from Kubuqi Desert (39° 40.198′ N, 110° 8.153′ E) in Inner Mongolia, northern China. The soil sample was collected in September at an altitude of 1441 m. The temperature at the moment of sampling was about 20 °C; the soil moisture content was extremely low, below 0.5%; and the organic matter content was less than 0.1%. The pH of the soil sample collected was 8.7, and the salt content was 3.3%. We used sterilized sampling spoons to take samples at the sampling point, mixed them evenly, placed them in sterile sampling bags, and quickly transported them back to the laboratory at room temperature. Before being used for research, the samples were sieved through a 0.5 mm sieve and stored in a refrigerator at 4 °C. The desert soil sample (0.5 g) was suspended in 4.5 ml sterile water and then shaken at a speed of 200 rpm for 30 min under 30 °C to mix evenly. After that, the mixture was diluted to four gradients (10^−1^, 10^−3^, 10^−5^, 10^−7^); 200 μL of the diluted solution with a dilution factor of 10^−7^ was aspirated by a pipette and spread onto Luria–Bertani agar plates (containing, per liter, 10 g NaCl, 10 g peptone, 5 g bacto-yeast extract and 20 g agar, pH 7.2–7.4), followed by incubation at 28 °C for 3 days. An orange-pigmented colony, designated 4-30^T^, was isolated and subsequently purified on LB agar plates at 28 °C. The purified strain was cultured on LB agar plates at 28 °C for further taxonomic experiments and maintained both on LB slants at 4 °C and in suspensions with 20% (*v*/*v*) glycerol (Beijing Chemical Works, Beijing, China) at −20 °C for long-term preservation.

The reference strains of the genus *Planococcus*, *Planococcus wigleyi* Sa1BUA13^T^, *Planococcus donghaensis* DSM 22276^T^, *Planococcus versutus* L10.15^T^, *Planococcus halocryophilus* Or1^T^, *Planococcus salinarum* DSM 23820^T^, and *Planococcus citreus* DSM 20549^T^ were obtained from the Leibniz Institute-Deutsche Sammlung von Mikroorganismen und Zellkulturen (DSMZ) culture collection for phenotypic characterization, fatty acid analysis, and other analyses.

### 2.2. 16S rRNA Gene Sequencing and Phylogenetic Analysis

Genomic DNA of strain 4-30^T^ was obtained using TIANamp Bacteria Genomic DNA Extraction Kit (TIANGEN BIOTECH, Beijing, China) according to the manufacturer’s instructions. The 16S rRNA genes were amplified using the universal forward primer 27F (5’-AGAGTTTGATCCTGGCTCAG-3’) and universal reverse primer 1525R (5’-AGAAAGGAGGTGATCCAGCC-3’), as described previously [17]. The purified PCR product was cloned into the pEASY-T1 vectors (TransGen Biotech, Beijing, China) and transformed into Trans1-T1 phage-resistant chemically competent cells (TransGen Biotech) for sequencing at ORI-GENE (Beijing, China). The sequences obtained were quality-checked and assembled via the SeqMan program (DNASTAR software version 7.1.0). After sequencing, the 16S rRNA gene sequence was submitted to the GenBank database and compared with the closest phylogenetic neighbors available from the EzBioCloud database (http://www.ezbiocloud.net, accessed on 6 December 2025) [18]. The 16S rRNA gene sequences of the related bacterial strains were retrieved from the GenBank database. Phylogenetic analysis based on the 16S rRNA gene was performed by the software package MEGA 11 [19] after multiple alignments of the sequence data with the Clustal_X program [20]. Phylogenetic trees were reconstructed with the neighbor-joining (NJ) [21], maximum-likelihood (ML) [22], and minimum-evolution (ME) [23] methods. The topology of the phylogenetic tree was evaluated by the bootstrap resampling method of Felsenstein [24] with 1000 replicates. The evolutionary distances were estimated by the algorithm of Kimura’s two-parameter model and are in the units of the number of base substitutions per site [25].

### 2.3. Whole-Genome Sequencing and Analysis

Genomic DNA of strain 4-30^T^ was prepared using the liquid nitrogen grinding method according to the procedure of Marmur [26]. The draft genome sequencing of strain 4-30^T^ was conducted using the paired-end sequencing technology with both the PacBio RS II and the Illumina HiSeq systems and assembled by SPAdes genome assembler (version 3.5.0) at Sangon Biotech (Shanghai, China). The DNA G+C content was determined with the RAST server using the draft genome sequence [27]. The rRNA and tRNA genes of genome sequences were predicted using the Barrnap version 0.8 and the tRNA-scan-SE version 2.0. Genes were identified using Glimmer version 3.02, and the predicted protein-coding sequences were then annotated against the KEGG (Kyoto Encyclopedia of Genes and Genomes) [28], COG (Clusters of Orthologous Groups of proteins) [29], Swiss-Prot [30], Pfam (Protein Families) [31], and GO (Gene Ontology) [32] databases to assign or improve general function annotations.

### 2.4. DNA–DNA Hybridization and Genome-Based Phylogenetic Analysis

The available genomes of *Planococcus* species were retrieved from the National Center for Biotechnology Information (NCBI) database. The digital estimation of DNA–DNA hybridization (dDDH) between strain 4-30^T^ and the reference species was carried out with the Genome-to-Genome Distance Calculator (http://ggdc.dsmz.de, accessed on 6 December 2025), provided by the DSMZ website, and the values lower than the 70% cut-off point were used as a standard for species delineation [33]. The average nucleotide identity (ANI) values based on the blast algorithm (ANIb), MUM-mer ultra-rapid aligning tool (ANIm), and usearch algorithm (orthoANIu) were calculated using the JSpecies WS (https://jspecies.ribohost.com/jspeciesws/, accessed on 6 December 2025) [34]. The orthoANIu values were estimated using the EzGenome web service (http://www.ezbiocloud.net/tools/ani, accessed on 6 December 2025) [35]. Moreover, a phylogenetic tree of strain 4-30^T^ and related species based on the whole genome was constructed using a bioinformatics platform: Type (Strain) Genome Server (http://tygs.dsmz.de/, accessed on 6 December 2025) [36]. Genomes of closely related species were retrieved from the GenBank database.

### 2.5. Morphological, Physiological, and Biochemical Analyses

The cultural characteristics of strain 4-30^T^ were determined on LB agar plates, with subculturing at 28 °C for 3 days. The cell morphology of strain 4-30^T^ was observed by scanning electron microscope after incubation on LB agar medium at 28 °C for 3 days. Standard Gram staining was performed according to the method described by Gerhardt et al. [37] and observed using light microscopy (BH-2; Olympus). Motility was detected by the presence of turbidity throughout tubes containing semi-solid LB medium and was confirmed by the hanging-drop method, as described by Bernardet et al. [38]. The anaerobic growth test [39] was performed on LB agar plates, and 1 g pyrogallic acid and 2 mL 10% NaOH (*w*/*v*) (Beijing Chemical Works, Beijing, China) were added to the plate, which was then sealed with Vaseline, and growth was monitored for up to 2 weeks. Growth at different temperatures (−20, −10, −5, 0, 4, 10, 15, 20, 25, 28, 30, 32, 35, 38, and 40 °C) was determined by monitoring the OD_600_ of the culture broth during cultivation. The pH range for growth (pH 4.0–12.0, at intervals of 0.5 pH unit) was determined by using the appropriate biological buffer system described by Xu et al. [40]. NaCl tolerance was tested by growing the cells in LB supplemented with 0–10% (*w*/*v*) NaCl at increments of 1% for 7 days. Catalase activity was determined from the production of oxygen bubbles after the addition of a drop of 3% (*v*/*v*) H_2_O_2_, and oxidase activity was detected by the oxidation of 1% (*w*/*v*) tetramethyl-p-phenylenediamine [41]. Methyl red and Voges–Proskauer tests and assays for nitrate reduction, hydrolysis of casein, gelatin, starch, and Tweens 20, 40, 60, and 80 were performed according to methods described by Dong and Cai [42]. Then, an assessment of enzyme activity and other physiological and biochemical tests were performed. The experiments were carried out in triplicate.

### 2.6. Chemotaxonomic Characterization

Biomass for chemotaxonomic analysis was collected by centrifugation from cultures grown in LB medium in shake flasks for 7 days at 28 °C and then washed twice with distilled water. Fatty acid methyl ester mixtures were obtained from the cells by saponification, methylation, and extraction according to the methods of Kämpfer et al. [43], analyzed according to the standard protocol of the Sherlock Microbial Identification System (MIDI Sherlock software package, version 6.0), and identified using version 6.0 of the TSBA database [44]. This standardized cultivation and harvest procedure is widely adopted for reliable comparison of fatty acid profiles among bacterial taxa. Isoprenoid quinones were extracted from lyophilized cells, purified, and analyzed by HPLC using the menaquinones of the reference type strains as standards according to the method described by Collins [45]. Polar lipids were extracted from 100 mg freeze-dried cells and separated by two-dimensional TLC on 10 × 10 cm silica-gel plates (silica gel 60 F_254_, Merck, Darmstadt, Germany), with chloroform–methanol–water (65:25:4, *v*/*v*) as the first solvent and chloroform–acetic acid–methanol–water (80:15:12:4, *v*/*v*) as the second one [46,47]. Total polar lipids were detected by spraying with the phosphomolybdic acid solution followed by heating at 110 °C for 10 min. Aminolipids were detected by spraying with a 0.4% (*w*/*v*) solution of ninhydrin in butanol saturated with water, followed by heating at 105 °C for 10 min. Phospholipids were detected by spraying with the reagent of Dittmer and Lester.

## 3. Results and Discussion

### 3.1. Phylogenetic Characteristics

**Figure 1:**
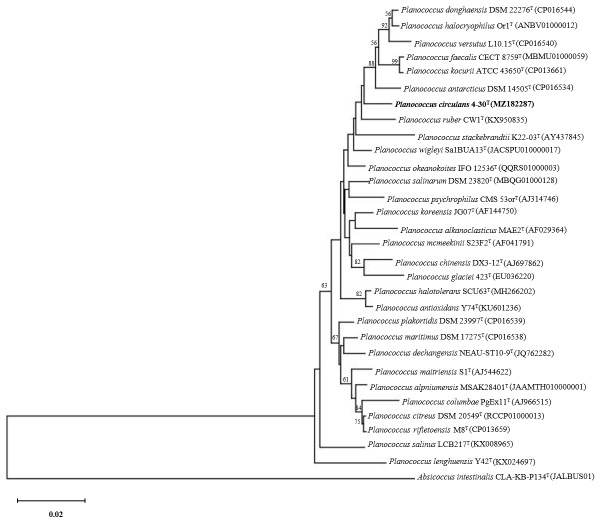
Neighbor-joining phylogenetic tree based on 16S rRNA gene sequences showing the relationships between strain 4-30^T^ and closely related species of the genus *Planococcus* within the family *Caryophanaceae*. Numbers at nodes represent bootstrap percentages (>50%) based on 1000 replicates. *Absicoccus intestinalis* CLA-KB-P134^T^ was used as an outgroup. Bar, 0.02 substitutions per nucleotide position.

Analysis of the 16S rRNA gene sequence of strain 4-30^T^ indicated that it was phylogenetically affiliated to the genus *Planococcus* and was closely related to *Planococcus wigleyi* Sa1BUA13^T^ (98.8%), followed by *Planococcus donghaensis* DSM 22276^T^ (98.5%), *Planococcus versutus* L10.15^T^ (98.4%), and *Planococcus halocryophilus* Or1^T^ (98.3%). In addition, analysis of the 16S rRNA gene sequence revealed that strain 4-30^T^ possesses signature nucleotides T and A at positions 183 and 190, respectively, which could distinguish it from the recognized species of the genus *Planomicrobium*. Because a comprehensive comparison across all currently recognized *Planomicrobium* species showed that all species in the genus consistently exhibit C and G at positions 183 and 190, which is different from strain 4-30^T^ (T183, A190). This distinct signature provides further molecular evidence supporting the differentiation of strain 4-30^T^ from its congeners and its belonging to the genus *Planococcus*. Figure 1 shows the positions of strain 4-30^T^ and its closest phylogenetic neighbors according to the NJ tree. The same relationships were obtained with phylogenetic reconstructions using ML (Figure S1, available in the online version of this article) and ME (Figure S2) methods.

### 3.2. Genome Features

**Figure 2:**
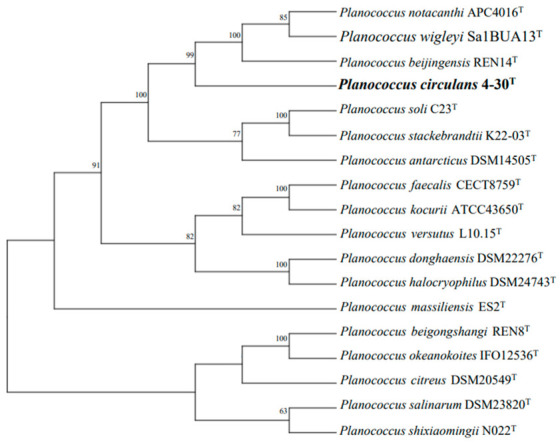
Whole-genome sequence tree generated with TYGS for strain 4-30^T^ and phylogenetically related type strains in the genus *Planococcus*. Tree inferred with FastME from GBDP distances calculated from genome sequences. Branch lengths are scaled in terms of GBDP distance formula d5. The numbers above branches are GBDP pseudo-bootstrap support values >60% from 100 replications.

The assembled genome sequence of strain 4-30^T^ has been deposited in the GenBank database under the accession number JAIQCL000000000. The draft genome of strain 4-30^T^ consisted of 3,917,538 bp in length and 74 contigs with the N50 value of 246,970 bp. The sequencing depth coverage was 285×. The DNA G+C content of strain 4-30^T^ was calculated directly from the genome sequence and determined to be 45.9% (WGS), within the range reported for species of the genus *Planococcus*. The detailed genomic features of 4-30^T^ are shown in Table S1 (available in the online version of this article). The genome had 3948 predicted protein-coding genes and 43 RNAs, of which 37 tRNAs and 6 rRNAs were predicted. Moreover, the dDDH values of strain 4-30^T^ with *P. wigleyi* Sa1BUA13^T^, *P. donghaensis* DSM 22276^T^, *P. versutus* L10.15^T^, and *P. halocryophilus* Or1^T^ were 26.7%, 22.6%, 21.8%, and 23.0%, respectively, and the dDDH value of strain 4-30^T^ with the type species of the genus *Planococcus*, *Planococcus citreus*, was 16.7%. To assess the genomic relatedness, we calculated pairwise Average Nucleotide Identity using three established algorithms: ANIb (using BLASTn, version 2.14.0), ANIm (using MUMmer, version 4), and OrthoANIu (using USEARCH, version 11.0.667). These methods were chosen as they provide complementary insights and are based on different calculation methods. ANIm is suitable for assessing average nucleotide consistency based on whole genome sequence alignment and has higher accuracy, while ANIb is suitable for processing large amounts of genomic data. It involves cutting a genome sequence into fixed-length segments and finally calculating the percentage of identity of all segments that can be aligned. In addition, when using OrthoANIu, only orthologous fragment pairs can be taken into consideration for calculating nucleotide identities. The ANIb, ANIm, and orthoANIu values between strain 4-30^T^ and the reference strains were 73.1–82.9%, 82.9–85.6%, and 73.8–83.3%. Therefore, the data obtained by different calculation methods and comparison strategies are different, which can comprehensively compare the average nucleotide similarity to understand the genomic structure and overall similarity relationship between strains. Obviously, the ANI and dDDH values are far below the commonly accepted cut-off values (ANI < 95–96%, dDDH < 70%) for species delineation [48], supporting the notion that strain 4-30^T^ is different from all closely related species described previously and represents a novel species within the genus *Planococcus* (refer to Table S2 for a list of all analyses performed on *Planococcus* type strains). The phylogenomic tree based on the genomes of strain 4-30^T^ and its closest relatives (with available genomes) is presented in Figure 2.

The comparison using different databases included 3948 genes in total; among them were 3044 protein-coding genes in the Swiss-Prot database, 3276 protein-coding genes in the Pfam database, 3398 protein-coding genes in the COG database, 2682 protein-coding genes in the GO database, and 2055 protein-coding genes in the KEGG database. According to the obtained annotation from the COG analysis, we found that 86.1% of protein-coding genes could be annotated to COG. Among the 3398 genes with known COG functional categories, 313 genes were related to amino acid transport and metabolism, accounting for 9.2% of the COG-annotated genes. This was followed by 257 genes (7.6%) related to carbohydrate transport and metabolism, and 234 genes (6.9%) related to inorganic ion transport and metabolism. To elucidate the genetic basis of salt tolerance in strain 4-30^T^, we screened its genome for putative stress-tolerant genes. Based on the genomes of strain 4-30^T^ according to COG annotation (E-value < 1 × 10^−5^), we analyzed the number of inorganic ion transport and metabolism gene clusters, and found that it contained genes related to the sodium–hydrogen exchanger. Genomic analysis based on COG annotation revealed that strain 4-30^T^ possesses two key genes involved in sodium homeostasis within the “inorganic ion transport and metabolism” category, and the locations were Scaffold1: 720013–721224 and Scaffold2: 377730–379763, which function as Na^+^/H^+^ antiporters. This may suggest a capacity for ion balance and pH regulation in fluctuating environments and further illustrates the fact that strain 4-30^T^, as a member of the genus *Planococcus*, is usually salt-tolerant. From the study, we found that Na^+^ could be pumped out of the cell through the sodium–hydrogen exchanger on the cell membrane to maintain the proper concentration of Na^+^, pH homeostasis, and ion dynamic balance, which provided a first impression of the salt tolerance of the strain. In addition, 48 stress-tolerant genes were identified in strain 4-30^T^ in the COG database, which could also help to explain the salt tolerance of strain 4-30^T^. Out of these, 32 genes were responsible for osmotic stress, 6 genes were identified as having a drug-resistance mechanism, and 8 genes were found to belong to the stress protein family. Among the 32 genes responsible for osmotic stress tolerance, 21 were identified as genes encoding ABC transporters related to inorganic ion transport and metabolism, which could regulate microbial membrane functions and inorganic ion transmembrane transport. Furthermore, a broader functional landscape was revealed through Gene Ontology (GO) annotation using the Blast2GO pipeline with standard parameters (E-value ≤ 1 × 10^−5^). Functional annotation of genes by comparison against the GO database revealed that there were 2107 genes related to molecular function, 1249 genes related to cellular components, and 1236 genes related to biological processes; the details of these genes are shown in Figures S3 and S4.

In addition, the antibiotic resistance genes and virulence genes of strain 4-30^T^ were predicted. The information on the antibiotic resistance genes of strain 4-30^T^ was obtained from the CARD database (http://arpcard.Mcmaster.ca, accessed on 5 December 2025). For the CARD database (version 1.1.3), RGI 5.2.1 software, including Perfect and Strict algorithms, was used as the screening criteria. Macrolide antibiotic resistance genes, tetracycline antibiotic resistance genes, and fluoroquinolone antibiotic resistance genes were predicted from the database. The antibiotics mentioned above were chosen for detecting antibiotic resistance, and the results indicated that strain 4-30^T^ could tolerate the selected antibiotics. Virulence genes of strain 4-30^T^ were annotated through the VFDB database (http://www.mgc.ac.cn/VFs/, accessed on 5 December 2025), and no gene related to known virulence was predicted from the databases mentioned above. In addition, it was found that there were three secondary metabolite synthesis gene clusters in the genome of strain 4-30^T^, two terpenes and one T3PKS, and the similarity between one terpene and the carotinoid gene cluster was 66%, suggesting that the bacterium could be used for industrial production in the pharmaceutical and food industries. In addition, through the annotation of carbohydrate active enzymes of strain 4-30^T^, it was found that both the GH35 family and GH42 family had *β*-galactosidase genes, which have potential application value in the food industry.

### 3.3. Morphological, Physiological, and Biochemical Characteristics

Strain 4-30^T^ was Gram-stain-positive, aerobic, motile, and catalase-positive, but oxidase-negative, which was similar to the closely related strains of the genus *Planococcus*. Colonies grown on LB agar plates for 3 days at 28 °C were orange-pigmented, circular, smooth, convex, and opaque. Growth of strain 4-30^T^ occurred at 4–38 °C (optimum, 25–28 °C), pH 6.0–11.0 (optimum, pH 9.0), and in the presence of 0–10% (*w*/*v*) NaCl (optimum, 1%). The physiological profile of strain 4-30^T^ reveals its adaptation to its desert isolation source. The broad salt tolerance range (0–10%) is likely a critical survival characteristic, enabling the strain to withstand periods of high salinity induced by extreme evaporation in surface soils, while the lower optimum salinity for growth suggests that its competitive advantage and active metabolic state are achieved under more moderate conditions, reflecting its adaptation to the desert ecosystem, which is a key characteristics of many microorganisms in fluctuating environments. Compared with its closest phylogenetic neighbors, strain 4-30^T^ had some different physiological and biochemical characteristics, such as being negative for hydrolysis of gelatin, which could distinguish strain 4-30^T^ from its closest phylogenetic relative *P. wigleyi* Sa1BUA13^T^. Other cultural, biochemical, and physiological characteristics of strain 4-30^T^, and the detailed comparison of selective characteristics between strain 4-30^T^ and closely related type strains, are summarized in Table 1 and in the species description.

### 3.4. Chemotaxonomic Characteristics

**Figure 3:**
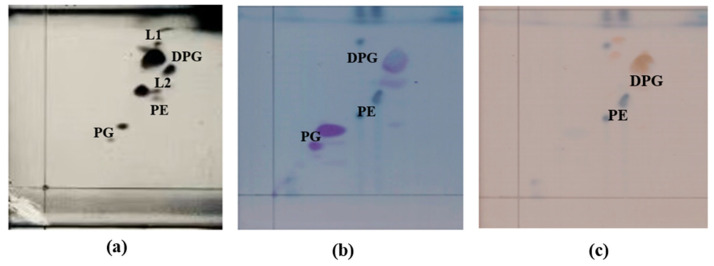
Cellular polar liquid profiles of strain 4-30^T^ after separation by two-dimensional TLC detected with ethanolic phosphomolybdic acid hydrate reagent (**a**), ninhydrin reagent (**b**), and molybdenum blue (**c**). Abbreviations: PE—phosphatidylethanolamine; DPG—diphosphatidylglycerol; PG—phosphatidylglycerol; L1–L2—unidentified phospholipids.

Under the standardized growth conditions described and comparable conditions, the cellular fatty acids of strain 4-30^T^ mainly consisted of iso-C_14:0_ (27.1%), anteiso-C_15:0_ (20.2%), C_16:1 _*ω*7*c* alcohol (18.7%), and iso-C_16:0_ (10.6%), consistent with other members of the genus *Planococcus*. Compared with the closely related strains within the genus *Planococcus*, strain 4-30^T^ contained 3.1% C_17:1_ *ω*9*c*, which was not found in other similar bacteria, and the contents of iso-C_14:0_ and C_16:1_ *ω*7*c* alcohol were notably higher than those in other reference strains. As detailed in Table 2, strain 4-30^T^ possessed a distinctive fatty acid profile when compared to its closest relatives. The predominant menaquinones in strain 4-30^T^ were MK-7 (54%) and MK-8 (46%), as in most other species of the genus *Planococcus*. The main polar lipids were diphosphatidylglycerol, phosphatidylglycerol, phosphatidylethanolamine, and two unknown lipids. (Figure 3). The chemotaxonomic characteristics of strain 4-30^T^ are consistent with its classification in the genus *Planococcus*.

### 3.5. Taxonomic Conclusion

Phylogenetic, phenotypic, and chemotaxonomic characteristics suggested that strain 4-30^T^ belonged to the genus *Planococcus*. Physiological and biochemical characteristics distinguished strain 4-30^T^ from the closely related type strains. Therefore, strain 4-30^T^ should be placed in the genus *Planococcus*, representing a novel species, for which the name *Planococcus circulans* sp. nov. is proposed.

## 4. Conclusions

Based on its phenotypic, phylogenetic, and chemotaxonomic characteristics, such as phylogenetic position, strain 4-30^T^ can be confirmed as a novel species within the genus *Planococcus*. The type strain 4-30^T^ (=CDMCC 1.2409^T^=KCTC 43405^T^) was isolated from a soil sample of the Kubuqi Desert in Inner Mongolia, China. The 16S rRNA gene and genome sequences of the type strain 4-30^T^ were deposited under the accession numbers MZ182287 and JAIQCL000000000, respectively.

### Description of Planococcus circulans *sp. nov.*

*Planococcus circulans* (cir’cu.lans. L. part. adj. *circulans*, making circles or round, circling).

Strain 4-30^T^ was positive for catalase, but negative for oxidase, nitrate reduction, methyl red, and Voges–Proskauer tests. Hydrolyses starch, Tween 20, Tween 40, and Tween 60, but not casein, cellulose, gelatin, urea, and Tween 80. Nitrate is reduced. Hydrolyzes aesculin, but not gelatin. Assimilates D-glucose, *N*-acetylglucosamine, D-mannitol, gluconic acid, malic acid, and maltose, but not L-arabinose, D-mannose, capric acid, adipic acid, citric acid, and phenylacetic acid as the sole source. Produces naphthol-AS-BI-phosphohydrolase, and valine arylamidase, but not alkaline phosphatase, arginine hydrolase, chymotrypsin, cystine arylamidase, acid phosphatase, esterase (C4), esterase lipase (C8), lipase (C14), leucine arylamidase, trypsin, urease, *α*-mannosidase, *α*-galactosidase, *α*-glucosidase, *β*-fucosidase, *β*-galactosidase, *β*-glucuronidase, *β*-glucosidase, and *N*-acetyl β-glucosaminidase.

This study fills a specific knowledge gap by providing a comprehensive taxonomic and functional description of a novel *Planococcus* species from an under-explored high salt desert. Prior to this work, the adaptive mechanisms of *Planococcus* species to the polyextreme conditions (high salt, aridity, UV) of inland deserts were speculative. In addition, the research on microorganisms in the desert mainly focuses on the phylum Actinobacteria, and there has been no study of the *Planococcus* species in the desert. Its distinct phylogenetic position and specific phenotypic profile expand the known ecological and genetic diversity within the genus, offering new insights for understanding microbial community assembly and resilience in desert ecosystems. This work fills a specific niche in our knowledge of desert *Planococcus* and provides a mechanistic framework for understanding bacterial adaptation to polyextreme environments.

In conclusion, we isolated a novel bacterium, designated as 4-30^T^, from the saline desert soil of the Kubuqi Desert. The aims of this study were to determine its precise taxonomic position using a polyphasic approach, elucidate its phenotypic and genotypic adaptive features, and propose it as the type strain of a novel species, *Planococcus circulans* sp. nov. In the future, we will conduct in-depth research on antibiotic resistance genes of the bacterial strain to verify whether it has the potential for application in the biodegradation of antibiotics and toxic substances.

## Figures and Tables

**Table 1:** Differential characteristics between strain 4-30^T^ and the closely related species of *Planococcus*. Strains: 1, 4-30^T^; 2, *Planococcus donghaensis* DSM 22276^T^; 3, *Planococcus versutus* L10.15^T^; 4, *Planococcus halocryophilus* Or1^T^; 5, *Planococcus salinarum* DSM 23820^T^; 6, *Planococcus citreus* DSM 20549^T^. All data are from this study except where otherwise indicated. +: positive; –: negative; w: weakly positive.

Characteristic	1	2	3	4	5	6
Colony color	Orange	Orange	Orange	Orange	Reddish-orange	Orange/yellow
Ranges for growth:						
Temperature (°C)	4–38	4–37	4–30	−10–37	4–38	4–37
NaCl (%, *w*/*v*)	0–10	0–12	0–15	0–19	0–13	0–10
pH	6.0–11.0	6.0–10.0	6.0–11.0	6.0–11.0	6.0–7.5	6.0–10.0
Hydrolysis of:						
Gelatin	–	–	–	+	–	+
Starch	+	+	+	–	–	–
Tween 80	–	–	–	+	+	–
Casein	–	+	–	–	+	–
Enzyme activity:						
Esterase (C4)	–	w	–	–	w	–
Valine arylamidase	+	–	–	–	–	–
*α*-Chymotrypsin	–	+	+	–	–	–
*β*-Galactosidase	–	w	–	–	–	+
*β*-Glucosidase	–	+	–	+	–	+
Alkaline phosphatase	–	+	–	–	–	–
Assimilation of:						–
D-Mannose	–	–	–	+	–	+
D-Maltose	+	+	–	+	–	+
*N*-acetylglucosamine	+	+	+	+	–	–
DNA G+C content (%)	45.9	47.0 *	39.4 ^§^	40.5 ^†^	44.8 ^‡^	48.5 ^&^

* Data from Choi et al. (2007) [14]. ^§^ Data from See-Too et al. (2017) [49]. ^†^ Data from Mykytczuk et al. (2012) [11]. ^‡^ Data from Yoon et al. (2010) [50]. ^&^ Data from Migula et al. (1894) [10].

**Table 2:** Fatty acid profiles of strain 4-30^T^ and the closely related species of the genus *Planococcus*. Strains: 1, 4-30^T^; 2, *Planococcus donghaensis* DSM 22276^T^; 3, *Planococcus versutus* L10.15^T^; 4, *Planococcus halocryophilus* Or1^T^; 5, *Planococcus salinarum* DSM 23820^T^; 6, *Planococcus citreus* DSM 20549^T^. Values are percentages of total fatty acids. The fatty acids in bold are the major cellular fatty acids (10% and above). All tests were carried out using similar methods. -: not detected; tr: trace amounts (<1.0%).

Fatty Acid (%)	1	2	3	4	5	6
Straight-chain fatty acids						
C_15:0_	3.5	1.7	1.2	tr	-	6.7
C_16:0_	1.2	6.3	4.0	6.8	-	-
C_17:0_	tr	1.3	tr	tr	-	2.3
Branched fatty acid						
iso-C_14:0_	**27.1**	3.4	3.4	2.2	4.2	8.8
iso -C_15:0_	1.8	4.7	1.9	2.5	2.7	-
iso-C_16:0_	**10.6**	9.4	5.5	4.9	7.5	8.1
iso-C_17:0_	1.0	6.4	1.9	3.6	2.5	-
iso-C_18:0_	tr	-	1.3	tr	-	-
anteiso-C_15:0_	**20.2**	**43.8**	**46.2**	**44.4**	**44.8**	**52.1**
anteiso-C_17:0_	1.1	**15.5**	**10.7**	**15.7**	**10.8**	5.2
Unsaturated fatty acids						
C_16:1 _*ω*7*c* alcohol	**18.7**	1.6	6.5	2.9	**16.7**	9.8
C_16:1 _*ω*11*c*	2.2	1.3	-	-	1.4	2.5
C_17:1 _*ω*9*c*	3.1	-	-	-	-	-
Mixed components						
Summed Feature 4 *	3.1	1.9	6.0	6.1	6.0	4.5

* Summed features are fatty acids that cannot be resolved reliably from other fatty acids using the chromatographic conditions chosen. The MIDI system groups these fatty acids together as one feature with a single percentage of the total. Summed feature 4 comprised iso-C_17:1_ I and/or anteiso-C_17:1_B.

## Data Availability

The GenBank/EMBL/DDBJ accession numbers for the 16S rRNA gene and the genome sequences of strain 4-30^T^ are MZ182287 and JAIQCL000000000, respectively, and other data are presented in the article and its Supplementary Materials.

## Supplementary Materials

The following supporting information can be downloaded at https://www.mdpi.com/article/10.3390/microorganisms14010231/s1: Figure S1: Phylogenetic tree constructed with the maximum-likelihood method based on 16S rRNA gene sequences of strain 4-30^T^ and other closely related species of the genus *Planococcus* within the family *Caryophanaceae*, showing the position of strain 4-30^T^ among its phylogenetic neighbors. *Absicoccus intestinalis* CLA-KB-P134^T^ was used as an outgroup. Numbers in notes represent bootstrap percentages (>50%) based on 1000 replicates; Figure S2: Phylogenetic tree constructed with the minimum-evolution method based on 16S rRNA gene sequences of strain 4-30^T^ and other closely related species of the genus *Planococcus* within the family *Caryophanaceae*, showing the position of strain 4-30^T^ among its phylogenetic neighbors. *Absicoccus intestinalis* CLA-KB-P134^T^ was used as an outgroup. Numbers in notes represent bootstrap percentages (>50%) based on 1000 replicates; Figure S3: Result of genes associated with general COG functional category prediction. On the left side is the distribution of the number of COG functional analyses. The abscissa represents the COG functional classification, and different colors and codes correspond to different types. The left vertical axis is the number of genes. On the right side of the figure is the legend, which gives the COG functional classification. Figure S4: GO gene annotation of strain 4-30^T^. The abscissa represents the three branches of GO, namely BP (biological process), CC (cellular component), and MF (molecular function), and further level 2 classification. The ordinate represents the proportion of genes; Figure S5: Transmission electron micrograph of cells of strain 4-30^T^ grown on LB medium for 3 days at 28 °C. Most of the cells were observed as single cocci. Bar, 0.2 μm; Table S1: General features of the genome of strain 4-30^T^; Table S2: Average nucleotide identity (ANIm, ANIb, and OrthoANIu), and digital DNA–DNA hybridization (dDDH) values (%) between strain 4-30^T^ (JAIQCL000000000) and the related strains of the genus *Planococcus*.
