# Supplementary material for: Planococcus circulans sp. nov., A Novel Bacterium Isolated from Kubuqi Desert Soil
Source: Microorganisms. 2026 Jan 19;14(1):231. doi: 10.3390/microorganisms14010231 (PMC12844253; doi:10.3390/microorganisms14010231)
Supplement: Supplementary file 1 [file microorganisms-14-00231-s001.zip › microorganisms-4058075-supplementary.pdf]

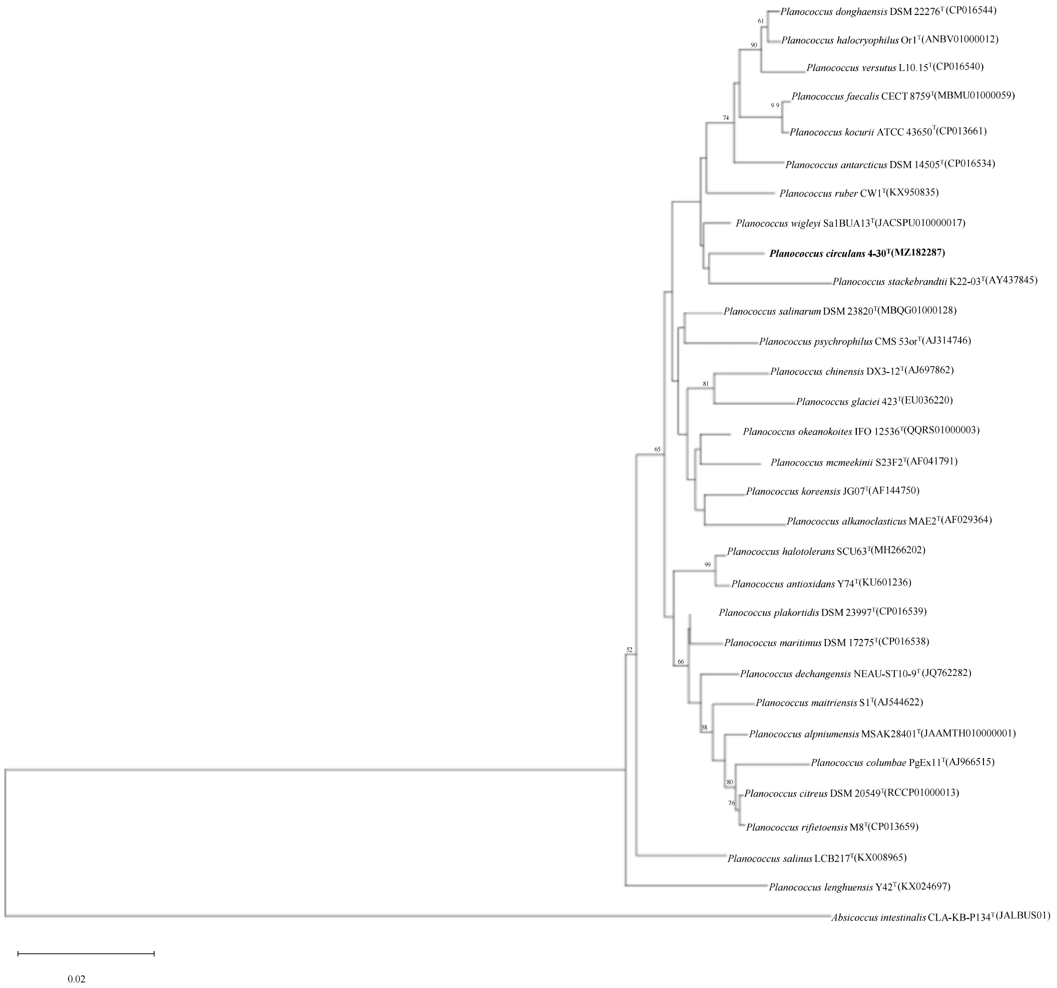

Fig. S1. Phylogenetic tree constructed with the maximum-likelihood method based on 16S rRNA gene sequences of strain 4-30^{T} and other closely related species of the genus *Planococcus* within the family *Caryophanaceae*, showing the position of strain 4-30^{T} among its phylogenetic neighbors. *Absicoccus intestinalis* CLA-KB-P134^{T} was used as an outgroup. Numbers at notes represent bootstrap percentages (>70%) based on 1000 replicates.

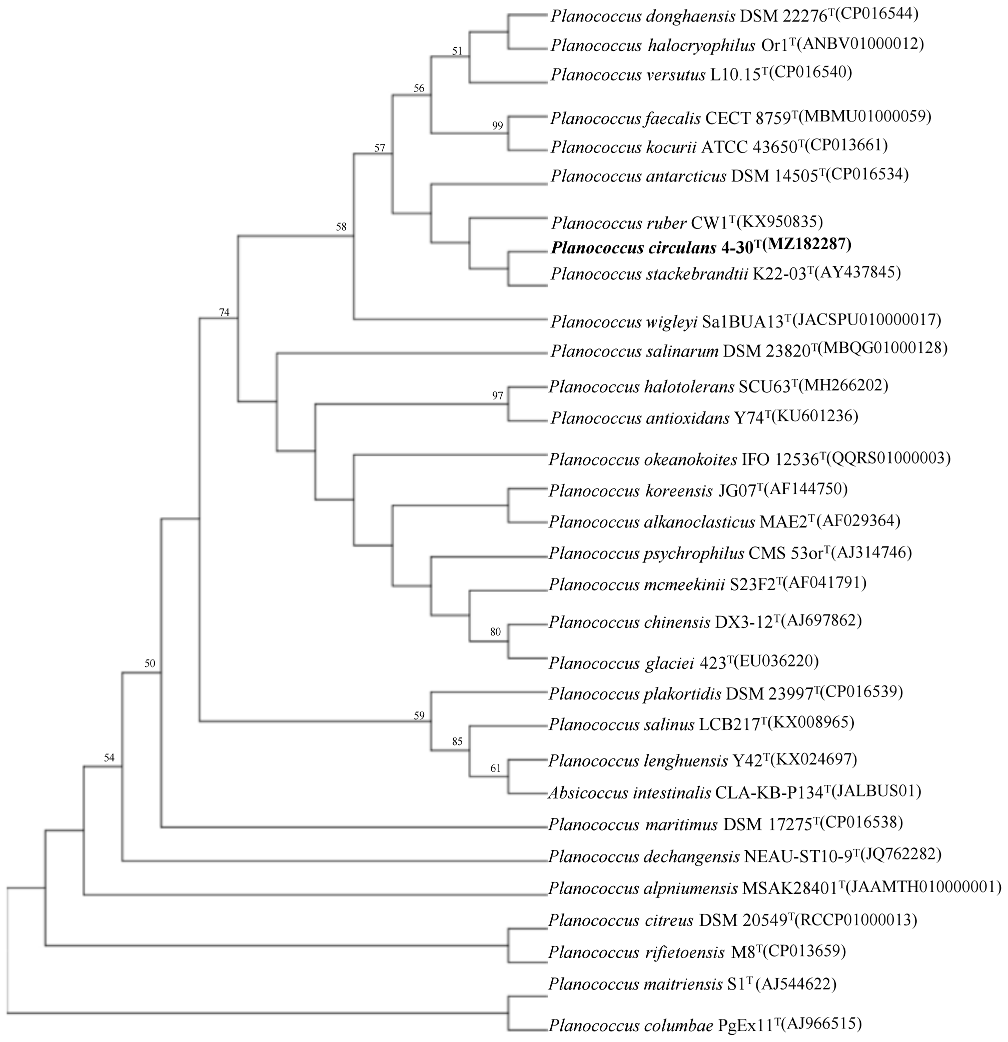

Fig. S2. Phylogenetic tree constructed with the minimum-evolution method based on 16S rRNA gene sequences of strain 4-30^{T} and other closely related species of the genus *Planococcus* within the family *Caryophanaceae*, showing the position of strain 4-30^{T} among its phylogenetic neighbors. *Absicoccus intestinalis* CLA-KB-P134^{T} was used as an outgroup. Numbers at notes represent bootstrap percentages (>70%) based on 1000 replicates.

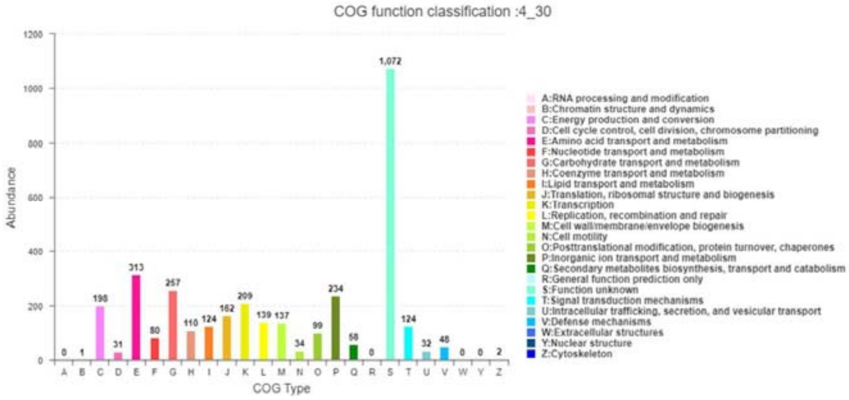

Fig. S3. Result of genes associated with general COG functional category prediction.

On the left side, it is the distribution of the number of COG functional analyses. The abscissa represents the COG functional classification that different colors and codes correspond to different types. The left vertical axis is the number of genes. On the right side of the figure is the legend, which gives the COG functional classification.

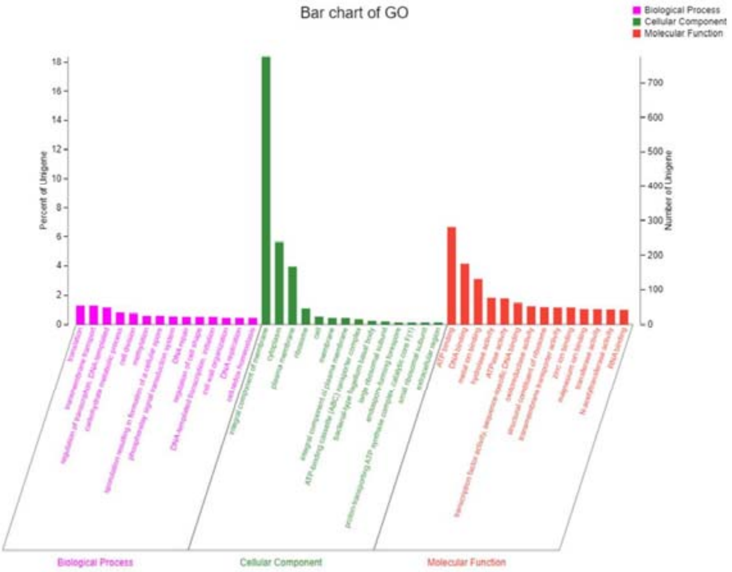

Fig. S4. GO gene annotation of strain 4-30^{T}. The abscissa represents the three branches of GO, namely BP (Biological Process), CC (Cellular Component), MF (Molecular Function), and further leve2 classification; the ordinate represents the proportion of genes.

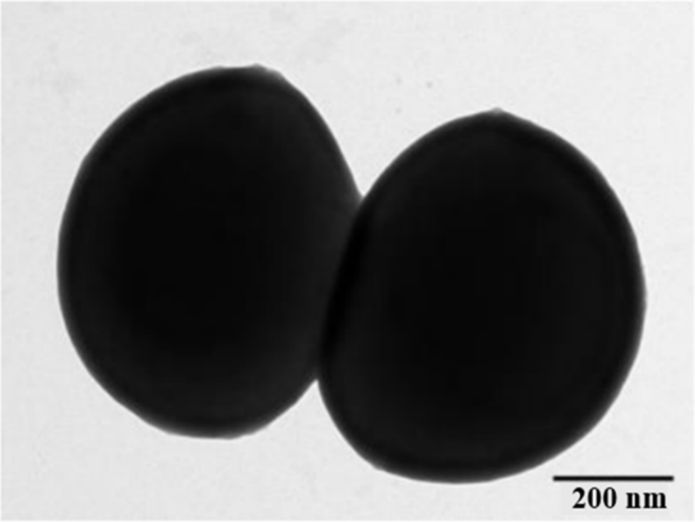

Fig. S5. Transmission electron micrograph of cells of strain 4-30^T grown on LB medium for 3 days at 28 °C. Most of the cells were observed as single cocci. Bar, 200 nm.

Table S1. General features of the genome of strain 4-30^T.

Parameter	Value
Genome size (bp)	3917538
Total genes	3948
Gene average length (bp)	852.95
Gene/Genome (%)	85.95
G+C content (%)	45.9
Scaffolds	48
No. of large scaffolds (>1000bp)	27
Scaffold N50 (bp)	308465
Scaffold N90 (bp)	90531
Contings	74
No. of large scaffolds (>1000bp)	39
Conting N50 (bp)	246970
Conting N90 (bp)	51273
No. of rRNA gene clusters	6
No. of tRNA clusters	37

Table S2. Average nucleotide identity (ANIm, ANIb and OrthoANIu), and digital DNA –DNA hybridization (dDDH) values (%) between strain 4-30^{T} (JAIQCL 000000000) and the related strains of the genus *Planococcus*.

Strain	Accession Number	dDDH (%)	ANIm (%)	ANIb (%)	OrthoANIu (%)
P. wigleyi Sa1BUA13^{T}	JACSPU010000017	26.7	85.6	82.9	83.3
P. donghaensis DSM 22276^{T}	CP016543	22.6	83.2	78.7	78.8
P. versutus L10.15^{T}	CP016540	21.8	82.9	77.9	78.1
P. halocryophilus Or1^{T}	CP016537	23.0	83.3	79.1	79.2
P. salinarum DSM 23820^{T}	VIFV00000000	20.0	83.9	73.8	79.5
P. chinensis DX3-12^{T}	JABWKT000000000	17.8	83.3	74.4	75.1
P. antarcticus DSM 14505^{T}	CP016534	26.3	84.8	82.1	82.5
P. citreus DSM 20549^{T}	RCCP00000000	16.7	82.9	73.1	73.8
